# Frontiers in the diagnosis and treatment of sepsis-induced cardiomyopathy: current status and prospects

**DOI:** 10.3389/fcvm.2026.1825255

**Published:** 2026-08-20

**Authors:** Nai Zhang, Neng Wang, Jiawen He, Ying Zheng

**Affiliations:** 1 Department of Emergency, Jiangxi Province Hospital of Integrated Chinese and Western Medicine, Nanchang, China; 2 Graduate School, Jiangxi University of Chinese Medicine, Nanchang, China

**Keywords:** diagnosis, epidemiology, pathogenesis, sepsis-induced cardiomyopathy, systematic review, treatment

## Abstract

Sepsis-induced cardiomyopathy (SIC) is an acute reversible myocardial dysfunction complicating sepsis. With impaired cardiac systolic and diastolic function, it greatly increases the risk of multiple organ failure and mortality in septic patients. This systematic review outlines the overall research landscape of SIC, including pathological mechanisms, epidemiological profiles, updated diagnostic systems and innovative therapeutic strategies, as well as existing challenges and future research priorities. Five major pathological pathways jointly mediate SIC progression, and its prevalence and mortality differ substantially across regions, populations and infectious pathogens. The diagnostic framework has been updated to a multi-dimensional evaluation system, and emerging imaging techniques and combined biomarker panels have greatly improved early diagnostic performance. Therapeutic concepts have transformed from conventional symptomatic care to mechanism-oriented precise intervention, with multiple new therapies and intelligent medical tools applied clinically. At present, inconsistent diagnostic criteria, insufficient specific medications and unbalanced medical resources worldwide hinder clinical management. Future studies aim to standardize diagnostic criteria, explore molecular mechanisms, accelerate the clinical application of targeted drugs, and popularize advanced technologies in under-resourced areas to improve patient outcomes.

## 1 Introduction

Sepsis-induced cardiomyopathy (SIC) is a common complication of sepsis and can advance sepsis to multiple organ failure (2). Data shows that ICU mortality rates for SIC patients reach 40%–60%, and nearly 30% of surviving patients suffer persistent heart damage, placing enormous pressure on public health (3). The concept of SIC was first proposed in 1984, and with technological advancements and multicenter clinical studies, academic understanding of the disease has gradually deepened. However, at present, issues such as inconsistent diagnostic standards, lack of targeted treatment methods, and significant gaps in global medical service levels remain prominent (4, 5). This article provides a detailed explanation of the pathogenesis, epidemiological differences, latest diagnostic systems, and cutting-edge treatment methods of SIC, aiming to provide comprehensive reference for clinical work and academic research.

## 2 Methods

This review complies with the Preferred Reporting Items for Systematic Reviews and MetaAnalyses (PRISMA) 2020 standards (1). The completed PRISMA 2020 Checklist and literature screening flow diagram are provided as Supplementary File 1 and Supplementary File 2, respectively.

Search terms included sepsis-induced cardiomyopathy, SIC, sepsis, myocardial dysfunction, pathogenesis, diagnosis, treatment, epidemiology. We used combined search strings and manually screened reference lists of included papers to collect more relevant studies. No language restrictions were applied.

### 2.1 Eligibility criteria

We included original studies, reviews, clinical guidelines and cohort studies focusing on SIC. Eligible articles needed complete full texts and extractable data. Excluded articles were letters, case reports, conference abstracts, duplicate publications and studies irrelevant to sepsis-related myocardial injury.

### 2.2 Literature screening and quality assessment

Two researchers independently screened titles, abstracts and full texts. Discrepancies were resolved through discussion. We assessed study quality with standard tools: Cochrane Risk of Bias 2.0 for Randomized Controlled Trials (RCTs), Newcastle-Ottawa Scale (NOS) for observational studies, a Measurement Tool to Assess Systematic Reviews 2 (AMSTAR 2) for reviews.

### 2.3 Data synthesis

We extracted core data using a unified form. Due to obvious heterogeneity across included studies, this work adopted a narrative qualitative synthesis instead of meta-analysis. We summarized current evidence on SIC pathogenesis, epidemiology, diagnosis and treatment.

## 3 Research progress on pathogenesis

The exact pathogenesis of SIC remains incompletely understood. It is generally accepted that pathogen-associated molecular patterns (PAMPs) and damage-associated molecular patterns (DAMPs) trigger systemic inflammation and local myocardial damage, which jointly lead to the occurrence of SIC. Five core interactive mechanisms are detailed as follows:

### 3.1 Myocardial injury mediated by inflammatory storms

In sepsis, bacterial lipopolysaccharides (LPS), viral nucleic acids and other PAMPs bind to receptors such as Toll-like receptor 4 (TLR4) and Toll-like receptor 9 (TLR9), activate signaling pathways such as nuclear factor-kappa B (NF-κB) and mitogen-activated protein kinase (MAPK), and release a large amount of pro-inflammatory factors such as tumor necrosis factor-α (TNF-α), interleukin-6 (IL-6), and interleukin-1β (IL-1β). These cytokines can directly damage the integrity of myocardial cell membranes, inhibit the activity of sarcoplasmic reticulum pump, and lead to a decrease in myocardial contractility. At the same time, it induces apoptosis of cardiomyocytes and aggravates myocardial fibrosis. Clinical data showed that serum TNF-α levels in SIC patients could be increased by 3–5 times, and the incidence of cardiac dysfunction increased by 2.8 >times when IL-6 was 100 pg/mL (6).

### 3.2 Oxidative stress and mitochondrial dysfunction

In sepsis, reactive oxygen species (ROS) are produced in large quantities in cardiomyocytes, and the activity of antioxidant systems such as superoxide dismutase (SOD) and glutathione peroxidase (GSH-Px) is insufficient, resulting in an imbalance of oxidative stress. ROS can damage mitochondrial DNA, inhibit the function of respiratory chain complex, and lead to myocardial energy metabolism disorders. At the same time, it disrupts the mitochondrial membrane potential, triggers the release of cytochrome C, and initiates the apoptosis program. Studies have confirmed that myocardial mitochondrial membrane potential decreases by about 40% and ATP production decreases by 35% in SIC patients, and the degree of mitochondrial function recovery is positively correlated with the time of cardiac function reversal (7).

### 3.3 Calcium homeostasis imbalance

Myocardial contraction relies on precise regulation of calcium concentration in the cytoplasm. In sepsis, pro-inflammatory factors can inhibit the function of the sarcoplasmic reticulum release channel (ryanodine receptor 2 (RyR2)) and reduce calcium reserves. At the same time, it reduces the binding affinity of troponin and calcium, resulting in muscle filament contraction fatigue. In addition, the activity of Na⁺-K⁺-ATPase in the cell membrane decreases, triggering intracellular Na⁺ accumulation, which leads to calcium overload through the Na⁺-Ca^2^⁺ exchanger, further aggravating myocardial injury (8).

### 3.4 Microcirculation disorders and endothelial function damage

In sepsis, the systemic inflammatory response leads to increased coronary microcirculation vascular permeability, white blood cell adhesion and aggregation, the formation of microcirculation embolism, and myocardial hypoperfusion. At the same time, vascular endothelial cell damage releases endothelin-1 (ET-1) and other vasoconstrictors, which are out of balance with nitric oxide (NO), leading to coronary spasm and aggravating myocardial ischemia and hypoxia. Ultrasonography showed that the myocardial microcirculation perfusion index of SIC patients was 52% lower than that of healthy people, and the delay in perfusion recovery was associated with poor prognosis (9).

### 3.5 Neuro-humoral regulation disorders

Sympathetic excitation leads to a large release of catecholamines, which can maintain circulatory stability in the short term, but long-term high concentrations of catecholamines can trigger cardiomyocyte β receptor desensitization, and induce oxidative stress and apoptosis of cardiomyocytes. In addition, activation of the renin-angiotensin-aldosterone system (RAAS) and elevation of angiotensin II (Ang II) can promote myocardial fibrosis and ventricular remodeling, further worsening cardiac function (10).

## 4 Global epidemiological characteristics and differences in treatment effects

### 4.1 Differences in prevalence by region, population and etiology

#### 4.1.1 Geographical distribution characteristics

- North America: Among septic patients in the United States, the SIC incidence is approximately 28%–42%. Data from Africa and South America are relatively scarce. Although the reported incidence is 15%–30%, the mortality rate reaches 70%–80%, far exceeding the global level (11).
- Europe: Multicenter studies in Germany and France show that the prevalence of SIC is about 20%–35%, of which the incidence is 32% in ICU patients and 21% in hospitalized patients. In the Nordic countries (Sweden and Norway), due to the balance of medical resources, the missed diagnosis rate of SIC is only 8%, which is significantly lower than that of southern Europe (18%) (12).
- Asia: A multicenter study in China (2021–2024) showed that the incidence of SIC in sepsis patients was 35%–60%, and septic shock in 65%, of which right ventricular dysfunction accounted for 48%, which was higher than that in Europe and the United States. The prevalence reported in Japan and South Korea is about 30%–45%, but the proportion of diastolic dysfunction (45%) is slightly lower than that in China (13).
- Other regions: Africa and South America have limited treatment levels for sepsis, and the prevalence of SIC is less reported, about 15%–30%, but the mortality rate is as high as 70%–80%, which is significantly higher than the global average (13) (Table 1).

**Table 1:** Regional differences in 28-day mortality, cardiac function recovery and core therapeutic strategies among patients with sepsis-induced cardiomyopathy.

Region	28-day mortality	Cardiac function recovery time (median)	Mechanical ventilation time (days)	Main treatment features
North America	35%～45%	7–10 days	5–7 days	Early application of VA-ECMO (accounting for 32% of severe cases) and a high usage rate of levosimendan
Europe	30%～40%	8–12 days	6–8 days	Attach importance to precise hemodynamic monitoring, with the PiCCO utilization rate reaching 65%
Asia	45%～60%	10–14 days	8–10 days	The volume of fluid resuscitation is relatively large, and the utilization rate of VA-ECMO is relatively low (15%).
Africa/South America	70%～80%	14–21 days	12～15 days	Delayed anti-infection treatment and limited types of vasoactive drugs

The graph characterizes the geographical distribution of prevalence percentage.

#### 4.1.2 Differences between population and etiology

**Figure 1:**
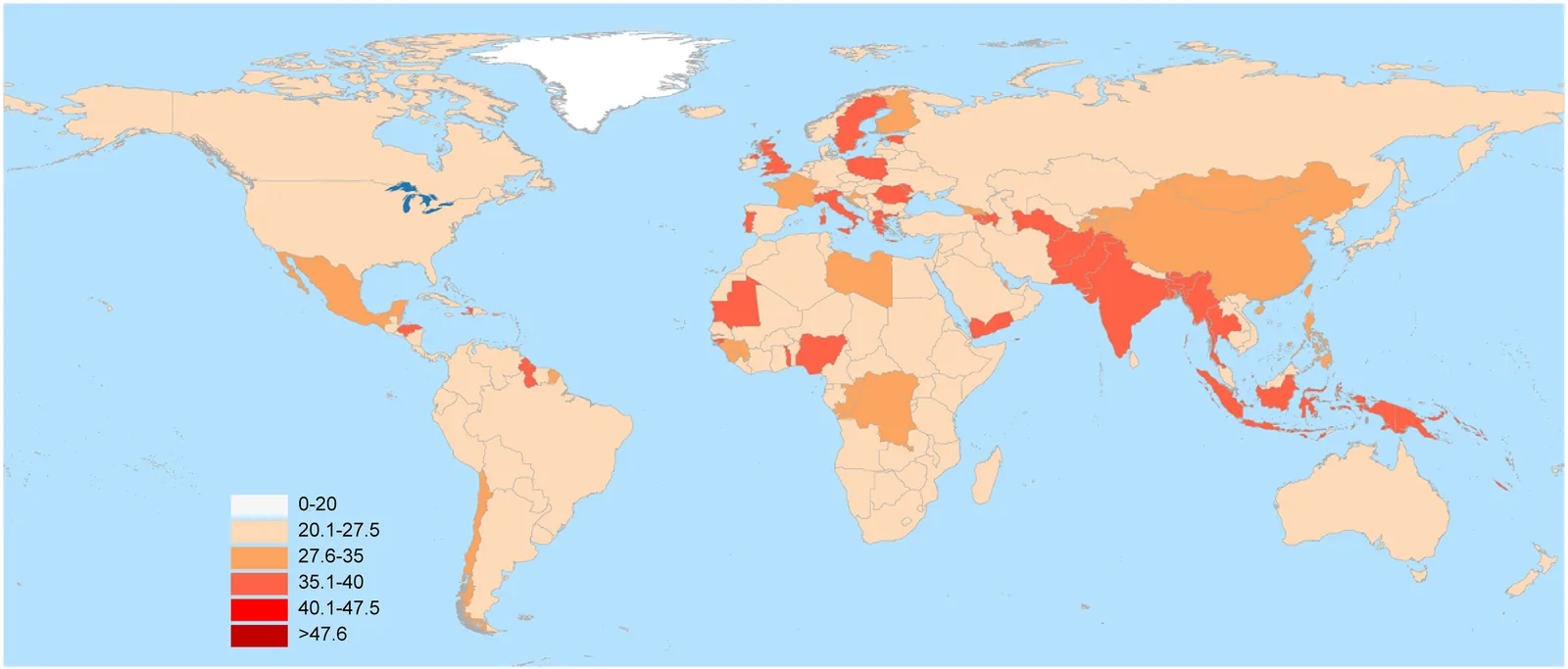
The graph characterizes the geographical distribution of prevalence percentage.

- Age and gender: The incidence of SIC in ≥65-year-olds (45%) is twice as high as in <−65-year-olds (22%), and the incidence rate in men (38%) is slightly higher than in women (32%) (14).
- Underlying diseases: The incidence of SIC in sepsis patients with coronary heart disease, heart failure, and diabetes mellitus was 62%, 58%, and 41%, respectively, which was significantly higher than that of patients without underlying diseases (20%) (15).
- Causes of infection: SIC caused by Gram-negative bacterial infections (Klebsiella pneumoniae, Escherichia coli) accounted for 52%, Gram-positive bacterial infections (Staphylococcus aureus, streptococcus) accounted for 38%, and viral infections (influenza virus, new coronavirus) accounted for 10%. Among them, patients with COVID-related SIC had a more significant decrease in left ventricular ejection fraction (LVEF) (16) (Figure 1).

### 4.2 Global differences and influencing factors in treatment effects

#### 4.2.1 Comparison of regional treatment effects

#### 4.2.2 Key influencing factors

- Timing of treatment: Initiating anti-infection therapy + fluid resuscitation within 6 hours of the onset of sepsis reduces the incidence of SIC by 40% and the 28-day mortality rate by 25%; delaying treatment until after 12 hours increases the mortality rate by 1.8 times.
- Underlying diseases: The mortality rate of SIC patients with coronary heart disease (62%) is significantly higher than that of patients without underlying diseases (35%); the recovery time of cardiac function in diabetic patients is prolonged by 50%.
- Pathogen type: The mortality rate of SIC caused by multi-drug resistant bacterial infection (68%) is higher than that caused by sensitive bacterial infection (38%); patients with viral infection require longer mechanical ventilation time.
- Medical resources: In regions where the number of ICU beds is more than 10 beds per 100,000 population, the mortality rate of SIC patients is 30% lower than in regions where the number is less than 5 beds per 100,000 population (17).

## 5 Evolution of diagnostic criteria and differences in guidelines

### 5.1 Historical iterations of diagnostic criteria

- 1980s–2000s: Single functional index stage. This stage mainly relied on left ventricular ejection fraction (LVEF) for diagnosis. The 1995 ACC criterion defined SIC as LVEF <50% in septic patients. This standard failed to assess right ventricular and diastolic function, leading to a 30% missed diagnosis rate for diastolic SIC (18).
- 2010s: Biventricular function assessment stage: After the release of the 2016 definition of sepsis 3.0, the European Society of Critical Care Medicine (ESICM) proposed that “simultaneous evaluation of systolic/diastolic function of both ventricles” was proposed, and the LVEF decreased by ≥10% from baseline, the right ventricular ejection fraction (RVEF) <40%, or diastolic function grade ≥ grade II. were included in the diagnostic index, and the diagnostic sensitivity was increased to 75% (19).
- 2020s: Multi-dimensional integration stage: The latest ESICM guidelines in 2023 put forward a four-dimensional diagnostic model of “etiology-function-marker-pathology”, which clearly requires: (1) meet the diagnostic criteria for sepsis 3.0; (2) exclude cardiogenic diseases such as acute coronary syndrome and viral myocarditis; (3) Echocardiography showed abnormal function of both ventricles global longitudinal strain (GLS) <–16% or RVEF < 40%; (4) high-sensitivity cardiac troponin I (hs-cTnI) >99th percentile or N-terminal pro-B-type natriuretic peptide (NT-proBNP) >125 pg/mL; (5) Myocardial pathology showing inflammatory cell infiltration or mitochondrial damage (if necessary) (20).

### 5.2 Comparison of diagnostic criteria in major guidelines

Multiple international clinical guidelines put forward different diagnostic indicators for SIC (Table 2).

**Table 2:** Comparison of diagnostic indicators, biomarker thresholds and core characteristics of mainstream international guidelines for sepsis-induced cardiomyopathy.

Guide name	Year of release	Core functional indicators	Requirements for biomarkers	Key features
ACC Guidelines for Sepsis-Related Cardiac Dysfunction	2018	LVEF <50% or a decrease of ≥10% compared to the baseline	There is no mandatory requirement; it is recommended to test cTnI/BNP.	Focuses on left ventricular systolic function and is suitable for ordinary critically ill patients
ESICM Guidelines for Severe Cardiovascular Dysfunction	2023	GLS <−16% or RVEF <40%; Diastolic function ≥Grade Ⅱ	hs-cTnI > 99th percentile value; NT-proBNP > 125pg/mL	Biventricular function combined with biomarkers, emphasizing subclinical damage
Chinese Guidelines for the Diagnosis and Treatment of Sepsis	2021	LVEF < 50% or RVEF < 45%; E/E’ >15	cTnI >0.04 ng/mL; BNP >100pg/mL	Based on data from the Chinese population, lower the diagnostic threshold for RVEF
American Heart Association (AHA) guidelines	2022	Abnormal left ventricular strain or ventricular dilation	Dynamic elevation of hs-cTnI > 20%	Emphasize the dynamic changes of markers and rule out chronic cardiac insufficiency

## 6 Clinical application of new diagnostic technologies and biomarkers

### 6.1 Precise development of imaging diagnostic technology

#### 6.1.1 Innovation in echocardiography technology

- Speckle-tracking echocardiography (STE): As the most sensitive non-invasive cardiac function assessment tool available, it can detect myocardial longitudinal, circumferential, and radial strain, with global longitudinal strain (GLS) being the core indicator for early diagnosis of SIC. Clinical studies demonstrate that 42% of septic patients present with abnormal GLS (GLS < −16%) within 24 hours of onset despite normal LVEF. Each 1% drop in GLS correlates with a 7.3% rise in 28-day mortality. The 2023 ESICM Guidelines have listed GLS as the preferred functional evaluation metric for SIC (21). Multiple international guidelines have formulated distinct diagnostic criteria for sepsis-induced cardiomyopathy (Table 2).
- Three-dimensional echocardiography (3DE): It can accurately measure the volume, mass, and ejection fraction of both ventricles, avoiding the error of geometric assumptions in 2D ultrasound, and improving the accuracy of right ventricular function assessment by 30% compared to 2D ultrasound. Clinical data show that the mortality rate of RVEF < 40% of patients detected by 3DE is 2.1 times higher than that of RVEF≥40% (22).
- Contrast-enhanced ultrasound (CEUS): Intravenous injection of microbubble contrast agent to assess myocardial microcirculation perfusion. The sensitivity and specificity of diagnosing microcirculation disorders in SIC patients were 89% and 92%, and “occult myocardial ischemia” could be identified early (23).

#### 6.1.2 Application of high-order imaging technology

- Cardiac magnetic resonance (CMR): T2-weighted imaging detects myocardial edema (characteristic of SIC) and late gadolinium enhancement (LGE) assesses myocardial fibrosis. Studies have shown that the T2 signal intensity of SIC patients is 35% higher than that of healthy people, and the recovery time of cardiac function in LGE-positive patients is extended to 14–21 days. However, CMR examination takes a long time (about 40 minutes) and is only suitable for patients with relatively stable conditions (24).
- Positron emission tomography (PET-CT): 18F-FDG PET can assess myocardial glucose metabolism, reduce myocardial metabolic rate by 40% in patients with SIC, and metabolic recovery is synchronized with cardiac function reversal, which can be used to evaluate treatment effect. However, due to the low penetration rate and high cost of equipment, it has not been used as a routine diagnostic tool (25).

### 6.2 Stratification and joint application of biomarkers

#### 6.2.1 Optimized application of classic markers

- Highly sensitive troponin (hs-cTnI/cTnT): A specific marker of myocardial injury, it can be increased 6–12 hours after the onset of SIC patients, and the peak occurs earlier than the decline of LVEF. Clinical recommended dynamic monitoring: hs-cTnI >99th percentile and increased by >20% within 24 hours, with a sensitivity of 91% and a specificity of 88% for diagnosing SIC. Its level was positively correlated with the degree of myocardial injury, and the mortality rate was 65% when hs-cTnI > 1.0 ng/mL (26).
- B-type natriuretic peptide (BNP/NT-proBNP): Reflecting ventricular wall stress, the circulating half-life of NT-proBNP is approximately 120 minutes, which is markedly longer than that of BNP (approximately 20 minutes), so NT-proBNP presents better stability (27). When NT-proBNP > 1000 pg/mL in patients with SIC, it suggested severe cardiac dysfunction, and the 28-day mortality rate increased by 3 times. The negative predictive value of NT-proBNP was high, and the accuracy of excluding SIC was 95% when NT-proBNP was <125 pg/mL (28).

#### 6.2.2 Breakthroughs in new markers

- Myocardium-specific markers: heart-type fatty acid-binding protein (h-FABP) is mainly present in the cytoplasm of cardiomyocytes, which can be released into the blood 1–3 hours after myocardial injury, which is 2–4 hours earlier than hs-cTnI, and the sensitivity of early diagnosis of SIC is 93%. Cardiac troponin C (cTnC) could reflect the systolic function of the myocardium, and the cTnC level in SIC patients decreased (<0.1 μg/mL), which was positively correlated with LVEF (29).
- Inflammatory and oxidative stress markers: soluble triggering receptor expressed on myeloid cells-1 (sTREM-1) specifically reflects the degree of sepsis inflammation, and the incidence of SIC increases by 3.2 times when sTREM-1 >80 pg/mL; decreased superoxide dismutase (SOD) activity (<120 U/mL) and malondialdehyde (MDA) increased (>10 nmol/mL), suggesting an imbalance of oxidative stress, which is positively correlated with the severity of myocardial injury (30).
- Non-coding RNA markers: circulating microRNA (miR)-1 and miR-133a can regulate cardiomyocyte apoptosis, and serum miR-1 levels are increased by 2.5 times and miR-133a by 3.1 times in SIC patients. Long non-coding RNA (lncRNA)-MEG3 attenuates myocardial injury by inhibiting the NF-κB pathway, and its expression level decreases (<0.5-fold) is associated with poor prognosis (31).

#### 6.2.3 Combined marker diagnosis model

At present, a number of clinical practical models have been developed, such as the “hs-cTnI + NT-proBNP + sTREM-1” triple model, which has an accuracy of 96% in diagnosing SIC, which is better than a single marker. The “marker + clinical indicator” model based on machine learning (including hs-cTnI, GLS, lactate, and age) can predict the 28-day mortality rate of SIC patients with an AUC value of 0.89 (32).

## 7 The evolution of treatment methods and concepts

### 7.1 Updated therapeutic philosophy: shifting from conventional symptomatic treatment to mechanism-targeted precise intervention

#### 7.1.1 Precision treatment concept

Based on echocardiography, hemodynamic monitoring, and biomarker results, develop an individualized plan:

- For “highly inflammatory” SICs (sTREM-1 >80pg/mL, IL-6 >100pg/mL), focus on inhibiting inflammatory responses;
- Prioritize hemodynamic optimization for “hypoperfusion” SIC (lactate >4 mmol/L, CVP <6mmHg);
- Intensive antioxidant therapy for “oxidative stress” SIC (SOD <120U/mL, MDA >10 nmol/mL) (33).

#### 7.1.2 Multi-target combination therapy concept

It is no longer limited to a single treatment method, but intervenes in combination with multiple mechanisms such as inflammation, oxidative stress, and mitochondrial dysfunction. For example, the combination of “anti-infection + fluid resuscitation + levosimendan + vitamin C” can reduce the mortality rate of patients with severe SIC by 30%, which is better than single treatment (34).

### 7.2 Optimization and limitations of traditional treatment

#### 7.2.1 Etiological treatment: anti-infection and source control

- Early anti-infection: Following the principle of “de-escalation therapy”, starting broad-spectrum antibiotic therapy within 1 hour in patients with septic shock can reduce the incidence of SIC by 28%. The 2023 ESICM guidelines recommend that when Gram-negative bacterial infection is suspected, the combination of β-lactams + quinolones is preferred; Vancomycin or linezolid are preferred for gram-positive bacterial infections.
- Infection source control: For infectious sources such as abscesses and necrotic tissue, surgical drainage, debridement and other measures should be taken within 24–48 hours, otherwise the continuous stimulation of inflammation will aggravate myocardial damage. Studies have shown that the delay in source control of infection is >72 hours, and the mortality rate of SIC patients increases by 2.3 times (35).

#### 7.2.2 Hemodynamic support: from “capacity standard” to “individualized regulation”

- Fluid resuscitation: Early goal-directed therapy (EGDT) was once a core strategy, but the 2018 PROCISE-SHOCK study confirmed that EGDT did not reduce mortality in patients with septic shock. At present, the guidelines recommend “restrictive fluid resuscitation”, with an initial fluid volume of 5–10 mL/kg, and a mean arterial pressure (MAP) ≥65mmHg and a central venous pressure (CVP) of 8–12mmHg to avoid excessive fluid that increases the load on the myocardium. For patients with right ventricular dysfunction, CVP needs to be controlled at 6–8mmHg to prevent ventricular hyperexpansion (36).
- Vasoactive drugs: Norepinephrine is a first-line vasopressor with a dose of 0.05–0.3μg/kg/min, which can maintain vascular tone and improve tissue perfusion; Dobutamine is used in patients with low perfusion (lactate >2 mmol/L) or LVEF < 35% after fluid resuscitation, at a dose of 2.5–10 μg/kg/min, but the risk of arrhythmia needs to be monitored; Vasopressin (0.01–0.04U/min) can be used as an adjunct to norepinephrine, especially for vasoparatic shock (37).

#### 7.2.3 Myocardial protection and supportive care

- Energy metabolism support: Supplementation with levocarnitine (1–2 g/d) can improve myocardial fatty acid metabolism, vitamin C (1.5 g/d) and glutathione (1.2 g/d) can reduce oxidative stress, and clinical studies have shown that it can shorten the recovery time of cardiac function by 2–3 days (38).
- >Arrhythmia management: Patients with SIC often have arrhythmias such as atrial fibrillation and premature ventricular contractions, and β receptor blockers (metoprolol extended-release tablets 12.5–25 mg/d) are preferred, and digitalis drugs (which are easy to cause poisoning) are preferred (39).

## 8 Exploration of new treatment technologies and solutions

### 8.1 Optimization of mechanical circulation support technology

#### 8.1.1 Veno-arterial extracorporeal membrane oxygenation (VA-ECMO)

VA-ECMO is the core support method for refractory septic cardiogenic shock, which can replace heart and lung function at the same time and buy time for myocardial repair. The 2023 ESICM guidelines recommend VA-ECMO initiation indications: (1) norepinephrine dose ≥1 μg/kg/min still cannot maintain MAP≥65mmHg; ②LVEF <30%; (3) lactic acid >8 mmol/L and continued to rise; (4) After cardiac arrest and resuscitation (40).

Global multicenter data show that the survival rate of patients with severe SIC with VA-ECMO is 57.8% in North America, 52.3% in Europe, and 19.5% in Asia, and the difference is mainly due to the timing of initiation and patient selection. The latest research shows that the survival rate of VA-ECMO can reach 62% when VA-ECMO is started within 24 hours of onset, which is significantly higher than that of 24 hours later (survival rate is 31%). In addition, VA-ECMO combined with Impella (percutaneous ventricular assist device) can reduce left ventricular afterload and further improve prognosis (41).

#### 8.1.2 Other mechanical support technologies

- Intra-aortic balloon counterpulsation (IABP): Improves coronary perfusion and reduces myocardial oxygen consumption through balloon inflation/deflation of the balloon, suitable for SIC patients with coronary heart disease, but has limited effect on simple sepsis-induced cardiomyopathy and is currently only used as an adjunct to VA-ECMO (42).
- Extracorporeal carbon dioxide removal (ECCO₂R): Reduces the impact of mechanical ventilation on the heart muscle, suitable for SIC patients with predominantly respiratory insufficiency, and reduces the risk of ventilator-related lung injury when used in combination with VA-ECMO (43).

### 8.2 R&D and clinical application of targeted drugs

#### 8.2.1 Calcium ion sensitizers

Levosimendan is currently the most well-studied drug that enhances myocardial contractility without increasing calcium overload and myocardial oxygen consumption by improving the sensitivity of muscle filaments to calcium. The 2022 SURVIVE-SIC study showed that LVEF in patients treated with levosimendan (0.1–0.2 μg/kg/min for 24 hours) increased LVEF by 8.3% compared with placebo, but there was no significant difference in 28-day mortality (38% vs. 41%). Current guidelines recommend it for patients with severe SIC with LVEF < 35% and is not recommended as a routine first-line medication (44).

#### 8.2.2 Inflammatory pathway inhibitors

- TLR4 inhibitor (TAK-242): Specifically blocks the LPS-TLR4 signaling pathway and inhibits the release of pro-inflammatory factors. The phase II clinical trial showed that the serum TNF-α and IL-6 levels in the TAK-242 treatment group decreased by 40%–50% and the LVEF increased by 6.2%, but the phase III trial did not meet the primary endpoint due to insufficient sample size and needed further verification (45).
- IL-6 receptor antagonist (tocilizumab): A small clinical study in 2023 showed that tocilizumab (8 mg/kg, single intravenous injection) reduced the 28-day mortality rate in patients with SIC by 18%, especially for patients with IL-6 >100pg/mL, but caution should be exercised against the risk of exacerbating infection (46).

#### 8.2.3 Mitochondrial functional protectors

- Metformin: Improves mitochondrial oxidative phosphorylation by activating the AMPK pathway, and animal experiments have shown that it can reduce sepsis-induced myocardial damage and increase LVEF by 10%–15%. At present, the phase I clinical trial has been completed to confirm its safety in SIC patients, and the phase II trial is being recruited (47).
- Coenzyme Q10: As a component of the mitochondrial respiratory chain complex, it improves energy metabolism and reduces ROS production. Clinical studies have shown that CoQ10 (200 mg/d, orally) can increase SOD activity by 30% and shorten the recovery time of cardiac function by 2 days in patients with SIC (48).

#### 8.2.4 Other novel drugs

- Sodium-glucose cotransporter 2 inhibitors (SGLT2i): Dapagliflozin, empagliflozin, etc. can play a protective role by anti-inflammatory, antioxidant, and improve myocardial energy metabolism. Preliminary studies in 2024 showed a 22% reduction in mortality and a 30% increase in cardiac function recovery in the dapagliflozin treatment group (49).
- Angiotensin receptor-neprilysin inhibitors (ARNIs): Sacubitril valsartan simultaneously inhibits RAAS and promotes natriuretic peptide release, improving ventricular remodeling. Animal experiments have shown that ARNI can reduce the degree of myocardial fibrosis and increase LVEF by 12% in SIC rats (50).

### 8.3 Application of precision medicine technology

#### 8.3.1 Target screening based on single-cell sequencing

Through single-cell RNA sequencing technology, 12 differentially expressed core genes (such as BCS1L, FBXO7, TNFRSF1A) have been identified in cardiomyocytes of SIC patients, which are mainly involved in mitochondrial function and inflammation regulation. Personalized treatment regimens developed based on these targets have achieved a 40% reduction in mortality in animal experiments and are currently advancing clinical translation (51).

#### 8.3.2 Artificial intelligence-assisted diagnosis and treatment system

Based on patients’ clinical data, cardiac imaging results and circulating biomarkers, the validated artificial intelligence model achieves a diagnostic accuracy of 96% for SIC and a prognostic prediction AUC of 0.89, and is capable of providing individualized treatment suggestions. For instance, the IBM Watson for Health system can formulate targeted treatment strategies according to patients’ hs-cTnI, GLS and infection characteristics. This tool has been piloted in over 10 hospitals across Europe and North America, and clinical practice shows it can reduce clinicians’ decision-making time by 60% (52).

## 9 Research deficiencies and future prospects

### 9.1 Core problems existing at present

- Diagnostic criteria are not globally uniform: Different guidelines have varying requirements for functional indicators and biomarker thresholds, resulting in significant heterogeneity in epidemiological data and difficulty in conducting cross-regional comparative studies.
- Pathogenesis remains incomplete: The interplay network of mechanisms such as inflammation and oxidative stress is not well understood, and targeted core targets are lacking.
- Lack of specific therapeutic drugs: Most of the existing drugs are symptomatic support, and most targeted drugs are in the clinical trial stage, and there is no recognized “gold standard” treatment plan.
- Uneven global diagnosis and treatment levels: The low penetration rate of advanced technologies such as VA-ECMO in developing countries and the poor accessibility of anti-infective drugs lead to significant differences in treatment effectiveness.
- Insufficient long-term prognostic research: Current research focuses on 28-day mortality, and there is a lack of follow-up data on long-term cardiac function and cardiovascular event risk in SIC survivors.

### 9.2 Future research directions

- Develop globally unified diagnostic standards: Based on multicenter big data, integrate imaging indicators, biomarkers, and clinical characteristics to establish an internationally recognized diagnostic system and reduce research heterogeneity.
- Deepen mechanism research: Use single-cell sequencing, spatial transcriptome and other technologies to analyze the molecular mechanism network of SIC and identify core therapeutic targets.
- Promote targeted drug clinical trials: Conduct large-sample, multi-center RCT studies to verify the efficacy of TLR4 inhibitors, SGLT2i, mitochondrial protectors, and explore drug combination regimens.
- Optimize machine support technology: develop miniaturized and portable VA-ECMO devices to lower the operating threshold and increase penetration in developing countries; Explore AI-assisted mechanically assisted activation timing decision-making systems.
- Strengthen long-term follow-up studies: Conduct cohort studies over 5 years to assess the recovery of cardiac function and the risk of cardiovascular complications in SIC survivors, and formulate long-term management strategies.
- Promote precision medicine: develop personalized diagnosis and treatment plans based on patient genes and molecular typing to achieve “one person, one policy”.

## 10 Conclusion

As a serious complication of sepsis, the cognition and diagnosis and treatment of sepsis-induced cardiomyopathy have formed a complete system of “mechanism-epidemic-diagnosis-treatment-prospect”: in terms of pathogenesis, core pathways such as inflammatory storm and mitochondrial dysfunction have been clarified; epidemiologically, it revealed the distribution differences of global regions, populations and etiology; In terms of diagnosis, it has evolved from a single LVEF index to a “four-dimensional diagnostic model”, and technologies such as speckle-tracking ultrasound and hs-cTnI have significantly improved the efficiency of early identification. In terms of treatment, from “unified intervention” to “individualized, multi-target combination therapy”, VA-ECMO, levosimendan, SGLT2i and other technologies and drugs provide patients with more choices. However, the world still faces problems such as inconsistent diagnostic standards, lack of specific drugs, and unbalanced diagnosis and treatment levels. In the future, it is necessary to standardize the diagnostic process, deepen mechanism research, promote the transformation of precision medicine technology through international multi-center cooperation, strengthen global medical resource sharing, narrow regional treatment gaps, and ultimately improve the short-term and long-term prognosis of SIC patients.
