# Supplementary material for: Frontiers in the diagnosis and treatment of sepsis-induced cardiomyopathy: current status and prospects
Source: Front Cardiovasc Med. 2026 Aug 20;13:1825255. doi: 10.3389/fcvm.2026.1825255 (PMC13538982; doi:10.3389/fcvm.2026.1825255)
Supplement: Supplementary file 1 [file Table1.docx]

| **Section and Topic** | **Item #** | **Checklist item** | **Location where item is reported** |
| --- | --- | --- | --- |
| **TITLE** | | |  |
| Title | 1 | Identify the report as a systematic review. | Title explicitly reflects the systematic review nature; Abstract and Introduction confirm the review type (e.g., "This systematic review summarizes the latest progress..."). |
| **ABSTRACT** | | |  |
| Abstract | 2 | See the PRISMA 2020 for Abstracts checklist. | Abstract follows PRISMA 2020 structure:  - Objectives: Summarize SIC pathogenesis/epidemiology/diagnosis/treatment progress.  - Methods: Systematic synthesis of public literature/guidelines.  - Results: Core mechanisms, regional disparities, diagnostic/treatment advances.  - Conclusions: Current gaps and future directions. |
| **INTRODUCTION** | | |  |
| Rationale | 3 | Describe the rationale for the review in the context of existing knowledge. | Introduction (Section 1): Highlights SIC’s role in sepsis-related multiple organ failure (ICU mortality 40%~60%) and long-term cardiac sequelae; notes unmet needs (inconsistent diagnostics, lack of specific drugs) to justify the review. |
| Objectives | 4 | Provide an explicit statement of the objective(s) or question(s) the review addresses. | Abstract and Introduction (Section 1): Objectives = Summarize SIC’s latest progress and challenges in pathogenesis, epidemiology, diagnosis, and treatment; Research questions = Clarify core mechanisms, global epidemiology, diagnostic evolution, and treatment advances. |
| **METHODS** | | |  |
| Eligibility criteria | 5 | Specify the inclusion and exclusion criteria for the review and how studies were grouped for the syntheses. | Data Availability & Supplementary File 1 (original):  - Inclusion: Peer-reviewed original studies, systematic reviews, clinical guidelines, multicenter trials (SIC patients, diagnostic/therapeutic outcomes).  - Exclusion: Case reports, non-peer-reviewed abstracts.  - Grouping: By region, age, pathogen, underlying disease for subgroup analyses. |
| Information sources | 6 | Specify all databases, registers, websites, organisations, reference lists and other sources searched or consulted to identify studies. Specify the date when each source was last searched or consulted. | Data Availability & Supplementary File 1 (original):  - Databases: PubMed, Embase, CNKI, Wanfang Data, VIP Chinese Science and Technology Periodical Database.  - Reference lists: Handsearched from included studies/reviews.  - Last search date: March 2025.  - Gray literature: Excluded. |
| Search strategy | 7 | Present the full search strategies for all databases, registers and websites, including any filters and limits used. | Supplementary File 1 (original):  - Keywords: ("Sepsis-induced cardiomyopathy" OR "SIC") AND ("pathogenesis" OR "epidemiology" OR "diagnosis" OR "treatment") AND "humans"[MeSH Terms].  - Filters: Human studies, English/Chinese, peer-reviewed.  - No start date limit. |
| Selection process | 8 | Specify the methods used to decide whether a study met the inclusion criteria of the review, including how many reviewers screened each record and each report retrieved, whether they worked independently, and if applicable, details of automation tools used in the process. | Supplementary File 1 (original):  - Two independent reviewers screened titles/abstracts and full-texts.  - Disagreements resolved by a third senior reviewer.  - Automation tools: EndNote (duplicate removal); 210 non-peer-reviewed records excluded via automation.  - PRISMA Flow Diagram (Supplementary File 2) details the process. |
| Data collection process | 9 | Specify the methods used to collect data from reports, including how many reviewers collected data from each report, whether they worked independently, any processes for obtaining or confirming data from study investigators, and if applicable, details of automation tools used in the process. | Supplementary File 1 (original):  - Two independent reviewers extracted data via a pre-piloted form.  - 10% of studies cross-checked for consistency (no major discrepancies).  - Missing data: Contacted 12 authors (3 provided data, 9 excluded).  - No automation tools used. |
| Data items | 10a | List and define all outcomes for which data were sought. Specify whether all results that were compatible with each outcome domain in each study were sought (e.g. for all measures, time points, analyses), and if not, the methods used to decide which results to collect. | Results (Sections 3–5) & Supplementary File 1 (original):  - Outcomes: Diagnostic accuracy (sensitivity/specificity), 28-day mortality, cardiac function recovery time, myocardial injury severity.  - All compatible results collected (time points: baseline to 28+ days). |
|  | 10b | List and define all other variables for which data were sought (e.g. participant and intervention characteristics, funding sources). Describe any assumptions made about any missing or unclear information. | Results (Section 3) & Supplementary File 1 (original):  - Variables: Study characteristics (design, sample size, region), participant characteristics (age, gender, underlying diseases), pathogen type, diagnostic criteria, intervention details, funding sources.  - Assumptions: Missing regional data attributed to low-resource limitations (noted in Discussion). |
| Study risk of bias assessment | 11 | Specify the methods used to assess risk of bias in the included studies, including details of the tool(s) used, how many reviewers assessed each study and whether they worked independently, and if applicable, details of automation tools used in the process. | Supplementary File 1 (original):  - RoB tools: ROBINS-I (observational studies), AMSTAR 2 (systematic reviews), AGREE II (guidelines).  - Two independent reviewers assessed RoB; disagreements resolved by a third reviewer.  - No automation tools used. |
| Effect measures | 12 | Specify for each outcome the effect measure(s) (e.g. risk ratio, mean difference) used in the synthesis or presentation of results. | Results (Sections 3–5):  - Diagnostic accuracy: Sensitivity, specificity, accuracy.  - Mortality: Percentage.  - Cardiac function recovery time: Median days.  - Myocardial injury: Biomarker levels (e.g., hs-cTnI), imaging indices (e.g., GLS).  - Narrative synthesis used due to heterogeneity. |
| Synthesis methods | 13a | Describe the processes used to decide which studies were eligible for each synthesis (e.g. tabulating the study intervention characteristics and comparing against the planned groups for each synthesis (item #5)). | Supplementary File 1 (original):  - Studies grouped by synthesis topic (pathogenesis/epidemiology/diagnosis/treatment) and subgroup (region/age/pathogen/underlying disease).  - Eligibility confirmed via cross-referencing with inclusion criteria (Item 5). |
|  | 13b | Describe any methods required to prepare the data for presentation or synthesis, such as handling of missing summary statistics, or data conversions. | Supplementary File 1 (original):  - Missing summary statistics: Addressed by author contact or study exclusion.  - No data conversions needed (consistent outcome reporting across studies). |
|  | 13c | Describe any methods used to tabulate or visually display results of individual studies and syntheses. | Main Manuscript & Supplementary Files:  - Tables: Regional treatment effects (Table 3.2.1), guideline comparisons (Table 4.2).  - Figures: Geographical prevalence distribution (Section 3.1.1), PRISMA Flow Diagram (Supplementary File 2). |
|  | 13d | Describe any methods used to synthesize results and provide a rationale for the choice(s). If meta-analysis was performed, describe the model(s), method(s) to identify the presence and extent of statistical heterogeneity, and software package(s) used. | Supplementary File 1 (original):  - Synthesis method: Narrative synthesis.  - Rationale: Heterogeneity in study designs, diagnostic criteria, and outcomes across regions.  - Meta-analysis: Not performed (insufficient homogeneity).  - No software used for synthesis. |
|  | 13e | Describe any methods used to explore possible causes of heterogeneity among study results (e.g. subgroup analysis, meta-regression). | Supplementary File 1 (original):  - Subgroup analyses: Conducted by region (North America/Europe/Asia/Africa), age (≥65/<65 years), pathogen type (Gram-negative/Gram-positive/virus), and underlying disease (coronary heart disease/diabetes).  - Meta-regression: Not performed (narrative synthesis). |
|  | 13f | Describe any sensitivity analyses conducted to assess robustness of the synthesized results. | Discussion (Section 8.1): Sensitivity analyses not performed due to narrative synthesis design; heterogeneity implications (e.g., regional diagnostic differences) noted as a limitation. |
| Reporting bias assessment | 14 | Describe any methods used to assess risk of bias due to missing results in a synthesis (arising from reporting biases). | Discussion (Section 8.1): No specific method used to assess reporting bias; limitation noted (e.g., underreporting of SIC in low-resource regions may skew epidemiological data). |
| Certainty assessment | 15 | Describe any methods used to assess certainty (or confidence) in the body of evidence for an outcome. | Discussion (Section 8.1): Certainty assessed based on study quality (low RoB in trials, moderate RoB in observational studies) and result consistency (e.g., consistent GLS diagnostic efficacy across studies). |
| **RESULTS** | | |  |
| Study selection | 16a | Describe the results of the search and selection process, from the number of records identified in the search to the number of studies included in the review, ideally using a flow diagram. | PRISMA Flow Diagram (Supplementary File 2) & Supplementary File 1 (original):  - 1582 records identified → 320 duplicates removed → 210 excluded (non-peer-reviewed) → 50 excluded (other reasons) → 992 screened → 785 excluded (irrelevant) → 207 full-texts retrieved → 12 unavailable → 195 assessed → 144 excluded → 51 studies included. |
|  | 16b | Cite studies that might appear to meet the inclusion criteria, but which were excluded, and explain why they were excluded. | Supplementary File 1 (original): 144 excluded studies listed with reasons (e.g., non-SIC focus, insufficient outcome data, low methodological quality); examples cited in the PRISMA Flow Diagram legend. |
| Study characteristics | 17 | Cite each included study and present its characteristics. | References (full list) & Supplementary File 1 (original): 51 included studies cited; characteristics summarized by type (23 multicenter trials, 18 observational studies, 5 systematic reviews, 5 guidelines), region, and publication year (82% post-2018). |
| Risk of bias in studies | 18 | Present assessments of risk of bias for each included study. | Supplementary File 1 (original): RoB summary table details assessments for each study; Section 8.1 summarizes RoB distribution (45% low RoB, 35% moderate RoB, 20% high RoB). |
| Results of individual studies | 19 | For all outcomes, present, for each study: (a) summary statistics for each group (where appropriate) and (b) an effect estimate and its precision (e.g. confidence/credible interval), ideally using structured tables or plots. | Results (Sections 3–5):  - Diagnostic outcomes: GLS sensitivity (89%), combined biomarkers accuracy (96%) (Section 5).  - Mortality: Regional rates (30%~80%) (Table 3.2.1).  - Recovery time: Median 7–21 days (Table 3.2.1).  - Structured tables (e.g., Table 4.2) present study-specific results. |
| Results of syntheses | 20a | For each synthesis, briefly summarise the characteristics and risk of bias among contributing studies. | Results (Sections 2–7):  - Pathogenesis synthesis: Based on 18 preclinical/observational studies (moderate RoB).  - Diagnosis synthesis: Based on 12 imaging/biomarker studies (low RoB).  - Treatment synthesis: Based on 23 multicenter trials (low RoB). |
|  | 20b | Present results of all statistical syntheses conducted. If meta-analysis was done, present for each the summary estimate and its precision (e.g. confidence/credible interval) and measures of statistical heterogeneity. If comparing groups, describe the direction of the effect. | Results (Sections 3–5): Narrative synthesis results presented (no meta-analysis); e.g., "Global SIC incidence 28%~60%", "VA-ECMO survival rate 57.8% (North America) vs. 19.5% (Asia)". Heterogeneity attributed to regional medical resources and diagnostic criteria. |
|  | 20c | Present results of all investigations of possible causes of heterogeneity among study results. | Results (Section 3): Subgroup analysis results presented; e.g., "≥65-year-olds have 2x higher SIC incidence than <65-year-olds", "Gram-negative infections account for 52% of SIC cases". |
|  | 20d | Present results of all sensitivity analyses conducted to assess the robustness of the synthesized results. | Not applicable (sensitivity analyses not performed; noted in Discussion Section 8.1). |
| Reporting biases | 21 | Present assessments of risk of bias due to missing results (arising from reporting biases) for each synthesis assessed. | Discussion (Section 8.1): Assessment presented (e.g., "Low-resource region underreporting may overestimate mortality differences"); no formal tool used. |
| Certainty of evidence | 22 | Present assessments of certainty (or confidence) in the body of evidence for each outcome assessed. | Discussion (Section 8.1): Certainty assessments presented; e.g., "High certainty in VA-ECMO efficacy (low RoB in trials)", "Moderate certainty in regional incidence data (moderate RoB in observational studies)". |
| **DISCUSSION** | | |  |
| Discussion | 23a | Provide a general interpretation of the results in the context of other evidence. | Discussion (Section 8.2): Results interpreted alongside recent reviews (e.g., Aissaoui et al., 2025; Sato et al., 2025); updated evidence (2023–2025) on SGLT2i and AI-assisted diagnosis integrated. |
|  | 23b | Discuss any limitations of the evidence included in the review. | Discussion (Section 8.1): Evidence limitations noted; e.g., "Few African/South American studies", "Inconsistent diagnostic criteria leading to epidemiological heterogeneity", "Most targeted drugs in early trials". |
|  | 23c | Discuss any limitations of the review processes used. | Discussion (Section 8.1): Review process limitations noted; e.g., "No meta-analysis due to outcome heterogeneity", "Gray literature excluded (potential missed data)", "RoB in observational studies may affect generalizability". |
|  | 23d | Discuss implications of the results for practice, policy, and future research. | Discussion (Section 8.2):  - Practice: Adopt 2023 ESICM four-dimensional model; prioritize early anti-infection.  - Policy: Improve VA-ECMO access in developing countries.  - Research: Focus on unified diagnostics and targeted drug trials. |
| **OTHER INFORMATION** | | |  |
| Registration and protocol | 24a | Provide registration information for the review, including register name and registration number, or state that the review was not registered. | Funding Declaration & Supplementary File 1 (original): No prior protocol registration; noted as a narrative systematic review (registration not mandatory). |
|  | 24b | Indicate where the review protocol can be accessed, or state that a protocol was not prepared. | Funding Declaration & Supplementary File 1 (original): No review protocol prepared (narrative systematic review). |
|  | 24c | Describe and explain any amendments to information provided at registration or in the protocol. | Not applicable (no registration/protocol). |
| Support | 25 | Describe sources of financial or non-financial support for the review, and the role of the funders or sponsors in the review. | Funding Declaration: No external financial support; funders had no role in study design, data synthesis, or manuscript writing. |
| Competing interests | 26 | Declare any competing interests of review authors. | Competing Interests: Authors declare no financial or non-financial competing interests. |
| Availability of data, code and other materials | 27 | Report which of the following are publicly available and where they can be found: template data collection forms; data extracted from included studies; data used for all analyses; analytic code; any other materials used in the review. | Data Availability: No original data generated; PRISMA Checklist (below) and PRISMA Flow Diagram (Supplementary File 2) are publicly available with the manuscript; cited databases/guidelines accessible via official URLs. |

*From:*  Page MJ, McKenzie JE, Bossuyt PM, Boutron I, Hoffmann TC, Mulrow CD, et al. The PRISMA 2020 statement: an updated guideline for reporting systematic reviews. BMJ 2021;372:n71. doi: 10.1136/bmj.n71. This work is licensed under CC BY 4.0. To view a copy of this license, visit <https://creativecommons.org/licenses/by/4.0/>
