# Supplementary material for: Frontiers in the diagnosis and treatment of sepsis-induced cardiomyopathy: current status and prospects
Source: Front Cardiovasc Med. 2026 Aug 20;13:1825255. doi: 10.3389/fcvm.2026.1825255 (PMC13538982; doi:10.3389/fcvm.2026.1825255)
Supplement: Supplementary file 2 [file Table2.docx]

**Identification of studies via databases and registers**

Records removed *before screening*:

- Duplicate records removed (n = 320)

- Records marked as ineligible by automation tools (n = 210) [e.g., non-peer-reviewed articles, conference abstracts]

- Records removed for other reasons (n = 50) [editorial letters, case reports, comments]

Records identified from*:

- Databases (n = 1582) [PubMed (n=620), Embase (n=513), Chinese Core Databases (CNKI, Wanfang, VIP) (n=449)]

- Registers (n = 0)

**Identification**

Records excluded** (n = 785) [excluded by two independent reviewers; no automation tools used for post-initial screening exclusion]

Records screened (n = 992) [1582 + 0 - 320 - 210 - 50]

Reports not retrieved (n = 12) [unavailable full-text, non-English language without translation]

Reports sought for retrieval (n = 207) [992 - 785]

**Screening**

Reports excluded (total n = 144):

Reason 1: Irrelevant to sepsis-induced cardiomyopathy (n = 63)

Reason 2: Not a primary study or systematic review (n = 41)

Reason 3: Lack of key outcomes (pathogenesis, diagnosis, treatment, epidemiology) (n = 28)

Reason 4: Duplicate reports of the same study (n = 12)

Reports assessed for eligibility (n = 195) [207 - 12]

Studies included in review (n = 51)

Reports of included studies (n = 51)

**Included**

Source: Page MJ, et al. BMJ 2021;372:n71. doi: 10.1136/bmj.n71.

This work is licensed under CC BY 4.0. To view a copy of this license, visit <https://creativecommons.org/licenses/by/4.0/>
